# Peak Systolic Velocity Measurements with Transcranial Doppler Ultrasound Is a Predictor of Incident Stroke among the General Population in China

**DOI:** 10.1371/journal.pone.0160967

**Published:** 2016-08-11

**Authors:** Hai-Bo Wang, Daniel T. Laskowitz, Jodi A. Dodds, Gao-Qiang Xie, Pu-Hong Zhang, Yi-Ning Huang, Bo Wang, Yang-Feng Wu

**Affiliations:** 1 Peking University Clinical Research Institute, Xueyuan Rd 38#, Haidian Dist, Beijing, 100191, China; 2 Department of Neurology, Duke University Medicine Center, Durham, North Carolina, 27710, United States of America; 3 The George Institute for Global Health at Peking University Health Science Center, Beijing, 100191, China; 4 Department of Neurology, Peking University First Hospital, Beijing, 100034, China; 5 Department of Neurology, Peking Union Medical Hospital, Beijing, 100730, China; 6 Department of Epidemiology and Biostatistics, Peking University School of Public Health, Beijing, 100191, China; Shanghai Institute of Hypertension, CHINA

## Abstract

**Background and Objective:**

It is necessary to develop an effective and low-cost screening tool for identifying Chinese people at high risk of stroke. Transcranial Doppler ultrasound (TCD) is a powerful predictor of stroke in the pediatric sickle cell disease population, as demonstrated in the STOP trial. Our study was conducted to determine the prediction value of peak systolic velocities as measured by TCD on subsequent stroke risk in a prospective cohort of the general population from Beijing, China.

**Methods:**

In 2002, a prospective cohort study was conducted among 1392 residents from 11 villages of the Shijingshan district of Beijing, China. The cohort was scheduled for follow up with regard to incident stroke in 2005, 2007, and 2012 by a study team comprised of epidemiologists, nurses, and physicians. Univariate and multivariate Cox proportional hazard regression models were used to determine the factors associated with incident stroke.

**Results:**

Participants identified by TCD criteria as having intracranial stenosis had a 3.6-fold greater risk of incident stroke (hazard ratio (HR) 3.57, 95% confidence interval (CI) 1.86–6.83, P<0.01) than those without TCD evidence of intracranial stenosis. The association remained significant in multivariate analysis (HR 2.53, 95% CI 1.31–4.87) after adjusting for other risk factors or confounders. Older age, cigarette smoking, hypertension, and diabetes mellitus remained statistically significant as risk factors after controlling for other factors.

**Conclusions:**

The study confirmed the screening value of TCD among the general population in urban China. Increasing the availability of TCD screening may help identify subjects as higher risk for stroke.

## Introduction

The Global Burden of Disease study in 2013 (GBD 2013) ranked stroke as the second most common cause of death and the third leading cause of life years lost worldwide, causing 110.1 deaths/100,000 persons and representing 12.5% of all-cause mortality [1]. Stroke age-standardized incidence has been declining in developed countries over the last 30 years, but there was a striking increase in developing countries, with approximately 70% of strokes occurring in developing nations [2]. In China, stroke remained the leading cause of death and became the second leading cause of adult disability [3]. Although a great many stroke prevention approaches have been implemented, the incidence and burden of stroke in China has been increasing over the past two decades [4], suggesting that primary prevention strategies are not well translated into practice [5]. Considering the limited resources in China, high-risk prevention strategies with ideal cost-effectiveness that are valid and suitable for identifying those at risk for stroke are needed. [6].

Transcranial Doppler ultrasound (TCD), which was introduced almost 30 years ago, has shown its potential screening value for stroke [7]. Ischemic stroke typically results from occlusion or stenosis of cerebral large arteries [8]. As blood flow velocity is inversely related to arterial diameter, intracranial arterial stenosis can be identified by an increase in velocity. In one study, the sensitivity was 91.6% and the specificity was 93.1% when diagnosing unilateral middle cerebral artery (MCA) stenosis by peak systolic velocities as detected by TCD. [9] As a non-invasive, safe, portable, relatively low-cost and well-tolerated technique, TCD is used for real-time evaluation of blood flow velocities in the major cerebral arteries and seems suited for screening high risk individuals among a large population. It has been confirmed by the Stroke Prevention Trial in Sickle Cell Anemia (STOP) that high flow velocities are correlated with subsequent stroke risk in children with sickle cell disease (SCD), and that TCD is a powerful predictor of stroke due to SCD [10, 11]. Even more important, STOP demonstrated that TCD identification of a high risk population and selecting patients for treatment (exchange transfusion) lowered the risk of stroke by 92%.

For TCD to be an effective and valuable screening tool in a large Chinese population, it should be demonstrated that an abnormal population as identified by TCD would be at increased risk for stroke. A prospective cohort study was undertaken to determine whether the peak systolic velocities detected by TCD can predict the subsequent stroke risk among habitants from the Shijingshan district of Beijing, China.

## Subjects and Methods

The study sample was taken from the cohort of the People’s Republic of China and the United States Collaborative Study of Cardiovascular & Cardiopulmonary Epidemiology. Participants’ characteristics, study goals, and design methods are described in detail elsewhere [12–14]. With the approval of the Cardiovascular Institute and Fuwai Hospital ethics committee, and the Peking University Health Science Center ethics committee, we conducted a prospective cohort study in the Shijingshan district of Beijing, China. In autumn 2002, 1392 subjects from 11 villages of the Shijingshan district were investigated to explore the effects of cerebral arterial peak systolic velocities on incident stroke. The information survey was considered as the baseline data of the cohort study. The cohort was scheduled for follow up in 2005, 2007, and 2012 respectively.

### Data collection at baseline

After obtaining written informed consent and following a 12 hour fast, participants were asked standardized questions about their demographics, past medical history, and classic cardiovascular risk factors, such as cigarette smoking, alcohol use, and degree of physical exercise. Based on the physical activity of reported occupation, participants were classified according to the International Standard Classification of Occupations (ISCO) [15] and merged into four groups with extremely low- (sedentary occupation including managers, scientists, office workers), low- (standing occupation without weight load including service workers, barber, salesperson), moderate- (technicians, machine operators) and high-intensity occupational activity (agricultural workers, craftsmen, labourers). Mean systolic and diastolic blood pressure (SBP and DBP) in sitting state (three measurements at 30 second intervals), body weight, and height were measured by one physician. Hypertension was defined as mean SBP ≥140 mmHg and/or DBP ≥90 mmHg, or as having used anti-hypertensive drugs in the past two weeks. Diabetic status was determined if the patient self-reported a history of diabetes mellitus, was using hypoglycemic drugs, or had a fasting blood glucose ≥7.0 mmol/L. Body mass index (BMI) was calculated as body weight divided by height squared (Kg/m^2^), and obesity was defined as BMI ≥28 Kg/m^2^. Myocardial infarction (MI) was diagnosed if there was a self-reported a history of MI or with specific findings on electrocardiogram, including acute ST-segment elevation, ischemic-type T wave changes, or necrotic Q waves.

Serum was collected from participants and sent to laboratory within three hours for assay. The concentrations of total cholesterol (TC) and fasting glucose levels were assessed by cholesterol oxidase and glucose oxidase methods, respectively. All of the tests were made daily by HITACHI 7020 Automatic Analyzer, which is assessed every 3 months. Hypercholesterolemia was defined as TC ≥200 mg/dL.

All of the participants were invited to undergo TCD examination carried out by a neurologist with experience in sonography. The equipment model, operation procedure, and images have been described in detail elsewhere [16, 17]. Briefly, peak systolic velocities of the bilateral extracranial (using 4 MHz probe) and intracranial arteries (using 2 MHz probe), including MCAs, anterior cerebral arteries (ACA), posterior cerebral arteries (PCA), common carotid arteries (CCA), internal carotid arteries (ICA), vertebral arteries (VA), and subclavian arteries, were measured among participants in supine resting positions.

### Follow-up of incident stroke

The cohort was followed up in 2005, 2007, and 2012 by a study team comprised of epidemiologists, nurses, and physicians. They investigated the past medical histories and reviewed the medical records from the interims at each follow-up. Strokes were confirmed by the presence of a focal neurological deficit of sudden or rapid onset lasting at least 24 hours based on clinical findings, or having had intracerebral hemorrhage, subarachnoid hemorrhage, cerebral thrombosis/embolism diagnosed by the participant’s general practitioner and/or based on computed tomographic scanning and/or magnetic resonance imaging. Undetermined participants were assessed independently by two neurologists, with a third neurologist available in cases of disagreement.

### Statistical analysis

Chi-square test or Fisher exact test were used to compare demographic characteristics of participants who were diagnosed as having intracranial vascular stenosis to those subjects without stenosis. In each patient, peak systolic velocities of individual arteries were recorded as the higher velocities on either side. Stenosis of an artery was diagnosed if the peak systolic velocity was ≥120 cm/s for an extracranial artery or ≥140 cm/s for an intracranial artery [18, 19]. Student's t-test was used to compare the difference in peak systolic velocities of each artery between participants with or without incident stroke. Stroke incidence density was calculated for participants who were stroke-free at baseline and completed at least one follow-up, dividing the number of incident strokes by the total number of person-years (PY) of follow-up. The follow-up time for each participant was calculated as the time between baseline survey and the last time follow-up or diagnosis date of stroke if confirmed. Poisson 95% confidence intervals (CI) were calculated for overall incidence density.

Univariate and multivariate Cox proportional hazard regression models were used to determine the factors associated with incident stroke and to calculate hazard ratios (HR) and corresponding 95% CI. Factors found significant in univariate analysis were included in a stepwise multivariate Cox proportional hazards regression model with entry criteria of *P*<0.20 and exit criteria of *P*>0.05. Statistical tests were performed using SAS 9.3 (SAS Institute, Inc, Cary, NC). All tests were 2-tailed and *P*<0.05 was considered the cut-off point for statistical significance for all analyses.

## Results

### Demographic characteristics of study participants

Between August and September 2002, a total of 1392 subjects were cumulatively recruited in the study. Of these 1392 participants, 63 participants were excluded because of a history of stroke and 4 were unable to complete TCD examination at baseline. Among the 1325 remaining participants, 62 (3.3%) failed to complete at least one follow-up, leaving a study sample of 1263 participants in the final cohort for analysis.

The cohort included 440 (34.8%) men and 823 (65.2%) women, and ages ranged from 42 to 73 years (median 56 years; interquartile range [IQR], 48–62 years). 56 (4.4%) subjects were diagnosed as having vascular stenosis (2.8% intracranial stenosis and 1.6% extracranial stenosis) by TCD examination at baseline. The baseline characteristics of all participants, with or without intracranial and extracranial stenosis, are shown in Table 1. Compared with subjects <50 years, subjects ≥60 years were significantly more linked to having intracranial/extracranial stenosis. More married participants had intracranial stenosis, but fewer married participants had extracranial stenosis. Those subjects with BMI ≥28 Kg/m^2^ had a higher prevalence of intracranial/extracranial stenosis, but without statistical significance.

**Table 1:** Demographic characteristics of participants with or without intracranial/extracranial stenosis at baseline in Shijingshan cohort (Beijing).

Demographic characteristics	Without intracranial and extracranial stenosis (N = 1207) N (%)	With intracranial stenosis (N = 36) N (%)	With extracranial stenosis (N = 20) N (%)	*P* value
Sex				
Male	423 (35.1)	13 (36.1)	4 (20.0)	0.3700
Female	784 (64.9)	23 (63.9)	16 (80.0)	
Age (Yrs)				
<50	358 (29.7)	6 (16.7)	3 (15.0)	0.0146
50–59	453 (37.5)	14 (38.9)	4 (20.0)	
≥60	396 (32.8)	16 (44.4)	13 (65.0)	
Marital status				
Married	1069 (88.6)	34 (94.4)	14 (70.0)	0.0189
Single, separated, divorced, or widowed	138 (11.4)	2 (5.6)	6 (30.0)	
Occupation				
Factory worker	227 (18.8)	3 (8.3)	1 (5.0)	0.1088
Homemakers	754 (62.5)	27 (75.0)	18 (90.0)	
Farmer	46 (3.8)	1 (2.8)	1 (5.0)	
Other	180 (14.9)	5 (13.9)	0 (0.0)	
Category of physical activity for occupation				
Extreme low	289 (23.9)	9 (25.0)	3 (15.0)	0.8617
Low	744 (61.6)	23 (63.9)	14 (70.0)	
Moderate	122 (10.1)	3 (8.3)	3 (15.0)	
High	52 (4.3)	1 (2.8)	0 (0.0)	
BMI (Kg/m^2^) ^ξ^				
<28	872 (72.2)	21 (58.3)	13 (65.0)	0.1502
≥28	335 (27.8)	15 (41.7)	7 (35.0)	

^ξ^ BMI: body mass index.

### Risk factors associated with incident stroke in univariate analysis

During the follow-up period from 2002 to 2012, 1263 participants were followed for an average of 9.16 (±2.27) years, for a total of 11569 PY. A total of 116 incident stroke cases were diagnosed, of which 101 (87.1%) were ischemic strokes, yielding an overall incidence of 1.00 per 100 PY (95% CI: 0.83–1.20).

Table 2 presents the demographic, behavioral, and health characteristics associated with stroke incidence in univariate analysis. Increased risk of incident stroke was associated with classic stroke risk factors: older age, cigarette smoking, hypertension, and diabetes mellitus. The participants who were homemakers had a 2.1-fold greater risk of incident stroke than factory workers. Interestingly, participants who exercised almost every day were more likely to have a stroke. It was shown by further analysis that participants who practiced more physical exercise were older and had higher prevalence of hypertension (S1 Table). We found no significant association between blood lipid TC level at baseline and the risk of stroke.

**Table 2:** Demographic, behavioral and health characteristics associated with the risk of incident stroke in Shijingshan cohort (Beijing) in univariate analysis.

Characteristics	HR (95% CI)	P value
Sex		
Male^§^	1.0	
Female	0.78 (0.54–1.14)	0.1974
Age (Yrs)		
<50^§^	1.0	
50–59	2.02 (1.11–3.66)	0.0216
≥60	4.11 (2.34–7.23)	< .0001
Marital status		
Married^§^	1.0	
Single, separated, divorced, or widowed	1.23 (0.71–2.11)	0.4588
Occupation		
Factory worker^§^	1.0	
Homemakers	2.15 (1.17–3.93)	0.0132
Farmer	1.63 (0.52–5.04)	0.5863
Other	1.46 (0.68–3.16)	0.1835
Category of physical activity for occupation		
Extreme low^§^	1.0	
Low	0.85 (0.56–1.29)	0.4433
Moderate	0.90 (0.46–1.75)	0.7471
High	0.52 (0.16–1.71)	0.2824
BMI (Kg/m^2^) ^ξ^		
<28^§^	1.0	
≥28	0.83 (0.54–1.26)	0.3688
Frequency of physical exercise		
Almost every day^§^	1.0	
1–4 times per week	0.27 (0.09–0.86)	0.0272
Hardly ever	0.66 (0.45–0.97)	0.0348
cigarette smoking		
No^§^	1.0	
Yes	1.63 (1.13–2.36)	0.0095
Drinking		
No^§^	1.0	
Yes	1.21 (0.81–1.80)	0.3511
Hypertension^£^		
No^§^	1.0	
Yes	2.75 (1.85–4.09)	< .0001
MI^¶^		
No^§^	1.0	
Yes	1.44 (0.20–10.31)	0.7147
Diabetes mellitus ^†^		
No^§^	1.0	
Yes	1.98 (1.17–3.35)	0.0115
TC (mg/dL) ^‡^		
<200^§^	1.0	
≥200	1.23 (0.85–1.77)	0.2703
TCD examination ^ψ^		
Normal^§^	1.0	
Intracranial stenosis	3.57 (1.86–6.83)	0.0001
Extracranial stenosis	1.87 (0.59–5.88)	0.2869

^§^ Reference group

^ξ^ BMI: body mass index.

^¶^ MI: myocardial infarction, self-reported a history of MI or with specific findings on electrocardiogram.

^†^ Diabetes mellitus: self-reported diabetes mellitus or a fasting blood glucose ≥7.0 mmol/L.

^£^ Hypertension was defined as a mean SBP of ≥140 mm Hg or a mean DBP of ≥90 mm Hg, or the use of antihypertensive drugs in the past 2 weeks.

^‡^ TC: total cholesterol.

^ψ^ TCD: Transcranial Doppler ultrasound.

### TCD examination results and association with incident stroke

Fifty-four (4.3%) of the 1263 participants had at least one MCA that was unable to be insonated, largely due to hyperostosis of temporal bone windows. Similarly, 54 (4.3%) and 18 (1.4%) participants had PCAs and ACAs, respectively, that could not be insonated due to the same technical limitation. The mean calculated of the peak systolic velocity measurements and the standard deviations of peak velocities of MCAs, ACAs, PCAs, and vertebral arteries were 95.35±22.37 cm/s, 80.36±20.46 cm/s, 44.46±9.99 cm/s and 51.76±17.35 cm/s, respectively. There were no statistically significant differences in peak systolic velocities in any artery between participants with or without incident stroke while using flow velocities as continuous variables in student's t-test (Table 3). However, the risk of incident stroke was significantly higher among those subjects with MCA peak velocities ≥ 140 cm/s (HR, 1.92; 95% CI, 1.01–3.67) and subjects with vertebral artery peak velocities ≥ 140 cm/s (HR, 14.19; 95% CI, 3.50–57.49) (Table 4).

**Table 3:** Peak flow velocity of extracranial artery and intracranial artery by subjects with or without incident stroke at baseline in Shijingshan cohort (Beijing).

	Subjects with incident stroke	Subjects without incident stroke	P value
common carotid artery	46.51±9.98	47.88±10.26	0.1699
external carotid artery	45.00±10.15	45.36±8.37	0.7128
internal carotid artery	50.69±11.19	51.28±11.24	0.5875
subclavian artery	72.50±18.89	73.65±16.46	0.5275
middle cerebral artery	96.44±26.26	95.24±21.95	0.6421
anterior cerebral artery	81.28±23.34	80.27±20.16	0.6621
posterior cerebral arteries	45.61±14.33	44.35±9.45	0.3574
vertebral artery	55.22±30.20	51.41±15.45	0.1819
basilar artery	57.32±20.69	56.46±16.56	0.6678

**Table 4:** The relationship between vascular stenosis and incident stroke based on individual artery.

Characteristics	HR (95% CI)
common carotid artery	
<120 cm/s	1.0
≥120 cm/s	NA
external carotid artery	
<120 cm/s	1.0
≥120 cm/s	NA
internal carotid	
<120 cm/s	1.0
≥120 cm/s	NA
subclavian artery	
<120 cm/s	1.0
≥120 cm/s	1.24 (0.46–3.35)
middle cerebral artery	
<140 cm/s	1.0
≥140 cm/s	1.92 (1.01–3.67)
anterior cerebral artery	
<140 cm/s	1.0
≥140 cm/s	1.97 (0.73–5.35)
posterior cerebral artery	
<140 cm/s	1.0
≥140 cm/s	NA
vertebral artery	
<140 cm/s	1.0
≥140 cm/s	14.19 (3.50–57.49)
basilar artery	
<140 cm/s	1.0
≥140 cm/s	3.78 (0.53–27.06)

**Fig 1:**
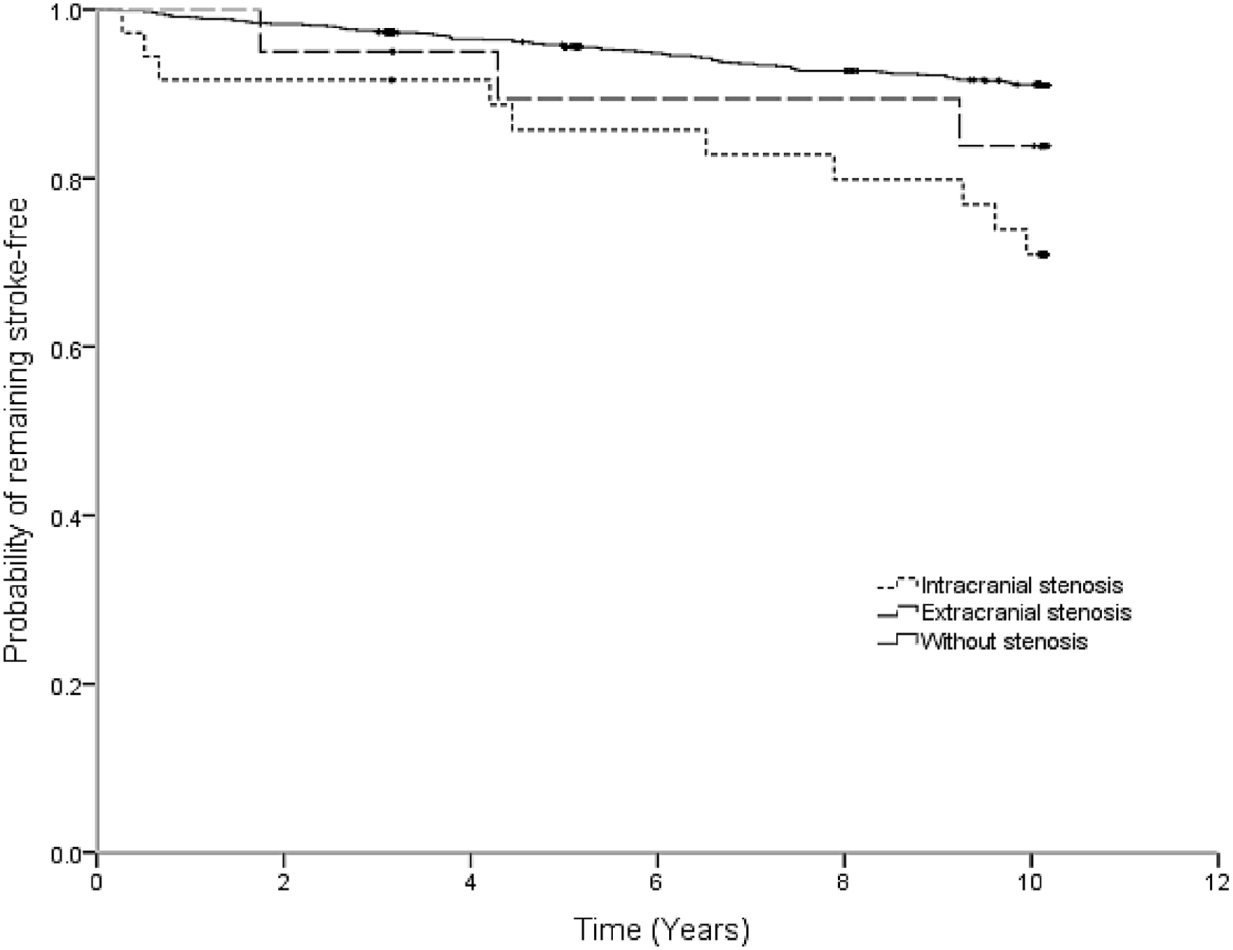
Kaplan-Meier Estimate of the probability of keeping stroke-free among participants with intracranial stenosis, extracranial stenosis or without stenosis in Shijingshan cohort (Beijing).

We found a significant association between vascular stenosis determined with the stated TCD criteria and incident stroke risk. Compared with participants without vascular stenosis, subjects with intracranial stenosis had a 3.6-fold greater risk of incident stroke (Table 2 and Fig 1). As shown in Fig 1, the participants with extracranial stenosis demonstrated a trend towards higher incidence of stroke after two years follow-up without statistical significance.

### Multivariate survival analyses

Table 5 shows adjusted HRs and 95% CIs of incident stroke in the multivariate Cox proportional hazards regression model. Overall, participants diagnosed by TCD criteria as having intracranial stenosis had a 2.5-fold greater risk of incident stroke than those without vascular stenosis after adjusting other risk factors or confounders; however, there was not a significant association between extracranial stenosis and the risk of incident stroke. Older age, cigarette smoking, hypertension, and diabetes mellitus remained statistically significant after controlling for other factors.

**Table 5:** Factors associated with incident stroke in Shijingshan cohort (Beijing) in multivariate analysis.

Characteristics	HR (95% CI)
50–59 Yrs old (vs. <50 yrs)	1.79 (0.98–3.28)
≥60Yrs old (vs. <50 yrs)	3.39 (1.92–6.01)
cigarette smoking	1.85 (1.27–2.68)
Hypertension^£^	2.38 (1.59–3.58)
Diabetes mellitus ^†^	2.11 (1.24–3.60)
Intracranial stenosis (vs. without stenosis)	2.53 (1.31–4.87)
Extracranial stenosis (vs. without stenosis)	0.98 (0.31–3.13)

^†^ Diabetes mellitus: self-reported diabetes mellitus or a fasting blood glucose ≥7.0 mmol/L.

^£^ Hypertension was defined as a mean SBP of ≥140 mm Hg or a mean DBP of ≥90 mm Hg, or the use of antihypertensive drugs in the past 2 weeks.

## Discussion

To our knowledge, this is the largest community-based study to explore the predictive value of TCD in the general population in China. Participants with intracranial stenosis as determined by TCD criteria demonstrated a higher risk of incident stroke. The findings of our study indicate that increased peak systolic velocities measured by TCD can identify the people at higher risk of stroke, and confirmed the potential screening value in the general population.

Significantly, the prospective cohort study found that participants with intracranial stenosis had a 2.5-fold greater risk of incident stroke than those without vascular stenosis (HR, 2.53; 95% CI, 1.31–4.87). Studies have shown that intracranial stenosis is the most common etiology of ischemic stroke in Asia [20, 21]. With the wide availability of non-invasive diagnostic tools such as TCD, intracranial artery stenosis can be detected in both asymptomatic individuals and in stroke patients [22, 23]. The “high risk” strategy for primary prevention of stroke, in which individuals at high risk are identified and targeted for preventive treatment, should be suited to the current situation in China, considering the limited medical resources and favorable effects of primary prevention methods. Identification and management of high risk individuals are the core foundation of the “high risk” strategy. Based on our findings, TCD would be a potentially valuable screening tool in this “high risk” strategy for two reasons. First, the portability, simplicity, safety, and non-invasive nature of the exam make TCD a logistically appropriate tool for diagnosis and monitoring of cerebral vascular stenosis while also remaining cost-effective [24]. Secondly, TCD has been validated for grading stenosis in other studies [25]. The sensitivity and specificity of the tool’s ability to identify intracranial vascular stenosis was 91.4% and 82.7% in one study compared with magnetic resonance angiography [18]. Although not as accurate as angiography (but also not posing the risks posed by this more invasive modality), TCD may be well-suited for screening among general population.

A relatively low prevalence (4.4%, 56/1263) of vascular stenosis was identified by TCD examination at baseline in our study. Previous studies have shown arterial stenosis diagnosed by TCD ranging from 5.9% to 12.6% among asymptomatic subjects [26–29]. The differences in the observed burden might have been resulted from sampling techniques, population heterogeneity (such as ethnicity, age and sex distribution), and different vascular risk factors in the source population.

A disadvantage of TCD is that it is highly operator-dependent due to absence of direct anatomical information about cerebral vasculature [7]. The study is more technically challenging to perform in patients with hyperostosis, and if the appropriate angle of insonation is not obtained, incorrect readings can be recorded. The application of this non-imaging technique requires a high level of experience and knowledge about cerebrovascular anatomy. Furthermore, there are great inherent variabilities of TCD signals resulted from the status of intracranial and extracranial arteries as well as systematic abnormalities [7].

As shown in other studies [30–33], we found similar risk factors or confounders significantly associated with incident stroke, including increasing age, cigarette smoking, hypertension, and diabetes mellitus. Hypertension has long been recognized as a global risk factor for stroke, with an estimated 51% of stroke deaths being attributable to high systolic blood pressure [30]. According to a new meta-analysis, subjects with blood pressure that is higher than the optimal 120/80 mmHg (prehypertension) were more likely to have a stroke [31]. It has been demonstrated in clinical trials that a blood pressure decrease can effectively reduce the risk of stroke [32].

Cigarette smoking is a major risk factor for the incidence of stroke in Chinese men. It should be noted that China is the largest producer and consumer of cigarettes in the world. A 10-year multi-centre prospective cohort study in China found that increased cigarette smoking and years of smoking was associated with increased incidence of stroke [33]. A cross-sectional study conducted with a nationally representative sample demonstrated that the clustering of cardiovascular and stroke risk factors is common in China [34]. With an aging population and rapid economic development, China is facing a great challenge in controlling chronic non-communicable diseases, such as cardiovascular disease and stroke, and more efforts should be given priority to the prevention of these conditions.

Interestingly, participants who exercised almost every day were more likely to have a stroke in the univariate analysis. It was speculated that the unexpected result was generated by confounding factors which were correlated with both physical exercise and incident stroke. It was confirmed by further analysis that participants who practiced more physical exercise were older and had higher prevalence of hypertension. Moreover, the significant relationship disappeared after adjusting other risk factors in the multivariate analysis.

Our study is subject to several limitations. First, only baseline TCD examination was done among all participants and the study was not repeated over the ten year period of follow-up. A change from normal TCD to abnormal TCD would be expected to occur in some participants if repeated TCD screening was carried out because we would expect vascular pathology to progress with aging. Repeated screening could potentially identify more people at risk for stroke. Second, the study was conducted in a cohort of habitants in Shijingshan county of Beijing, and our study setting may limit the generalizability of our results. However, the setting included access to comprehensive medical records and provided for a relatively long duration of follow-up. Third, the lower proportion of stenosis diagnosed by TCD may be due to one TCD peak systolic velocity measurement being recorded per vessel rather than calculating the mean from multiple readings over time. However, these limitations are partially overcome by the large study population, as well as the long follow-up period of 10 years. Fourth, only final diagnosis results of stroke (occurrence and the type of stroke) was collected by study team, without detailed information on imaging diagnosis. Therefore, it is impossible to report the number of incident stroke confirmed by imaging examination. Finally, no consensus criteria of transcranial Doppler velocities have been determined for the detection of threshold stenosis. Moreover, different arteries may have different optional values of velocity on TCD in detecting artery stenosis. For example, ≥140 cm/s in a PCA is more concerning than that in an MCA as the degree of stenosis would probably be more severe with a high velocity in a smaller vessel. However, the reference criteria (140 cm/s of peak systolic velocity) was determined by comparison with magnetic resonance angiography and a good diagnostic accuracy (sensitivity 90.3% and specificity 95.2%) was achieved [19].

## Conclusions

The study establishes that participants with high peak systolic flow velocities on TCD are at higher risk for incident stroke and confirmed the screening value of TCD among the general population in urban China. Screening with TCD may provide awareness of patients at higher risk for stroke and may prompt providers to act more aggressively with primary preventative care. Increasing the availability of TCD screening may help identify high risk subjects and prevent strokes among general population. However, the screening strategy should be further studied in the future, and generalizability can be determined by studying other populations internationally.

## Supporting Information

S1 File SAS database for data analysis of the research. (SAS7BDAT)

S1 Table The relationship between frequency of physical exercise and age, hypertension. Hypertension was defined as a mean SBP of ≥140 mm Hg or a mean DBP of ≥90 mm Hg, or the use of antihypertensive drugs in the past 2 weeks.
(DOCX)

## Abbreviations

TCD: Transcranial Doppler ultrasound
GBD: Global Burden of Disease
MCA: middle cerebral arteries
SCD: sickle cell disease
STOP: Stroke Prevention Trial in Sickle Cell Anemia
DBP: diastolic blood pressure
SBP: systolic blood pressure
BMI: Body mass index
MI: Myocardial infarction
TC: total cholesterol
ACA: anterior cerebral arteries
PCA: posterior cerebral arteries
CCA: common carotid arteries
ICA: internal carotid arteries
VA: vertebral arteries
PY: person-years
CI: confidence intervals
HR: hazard ratios
IQR: interquartile range

## References

[1] Mortality GBD, Causes of Death C. Global, regional, and national age-sex specific all-cause and cause-specific mortality for 240 causes of death, 1990–2013: a systematic analysis for the Global Burden of Disease Study 2013. Lancet. 2015;385(9963):117–71. 10.1016/S0140-6736(14)61682-2 25530442 PMC4340604

[2] Berkowitz AL. Stroke and the noncommunicable diseases: A global burden in need of global advocacy. Neurology. 2015;84(21):2183–4. 10.1212/WNL.0000000000001618 . 26009559

[3] Yang G, Wang Y, Zeng Y, Gao GF, Liang X, Zhou M, et al Rapid health transition in China, 1990–2010: findings from the Global Burden of Disease Study 2010. Lancet. 2013;381(9882):1987–2015. 10.1016/S0140-6736(13)61097-1 . 23746901 PMC7159289

[4] Liu L, Wang D, Wong KS, Wang Y. Stroke and stroke care in China: huge burden, significant workload, and a national priority. Stroke; a journal of cerebral circulation. 2011;42(12):3651–4. 10.1161/STROKEAHA.111.635755 . 22052510

[5] Zhao JJ, He GQ, Gong SY, He L. Status and costs of primary prevention for ischemic stroke in China. Journal of clinical neuroscience: official journal of the Neurosurgical Society of Australasia. 2013;20(10):1427–32. 10.1016/j.jocn.2013.01.012 . 23938016

[6] Feigin VL, Wang W, Fu H, Liu L, Krishnamurthi R, Bhattacharjee R, et al Primary stroke prevention in China—a new approach. Neurological research. 2015;37(5):378–80. 10.1179/1743132815Y.0000000025 25820024 PMC4462843

[7] Topcuoglu MA. Transcranial Doppler ultrasound in neurovascular diseases: diagnostic and therapeutic aspects. Journal of neurochemistry. 2012;123 Suppl 2:39–51. 10.1111/j.1471-4159.2012.07942.x . 23050641

[8] Rothman SM, Fulling KH, Nelson JS. Sickle cell anemia and central nervous system infarction: a neuropathological study. Annals of neurology. 1986;20(6):684–90. 10.1002/ana.410200606 . 3813497

[9] Wang L, Xing Y, Li Y, Han K, Chen J. Evaluation of flow velocity in unilateral middle cerebral artery stenosis by Transcranial Doppler. Cell biochemistry and biophysics. 2014;70(2):823–30. 10.1007/s12013-014-9986-4 . 24833432

[10] Adams R, McKie V, Nichols F, Carl E, Zhang DL, McKie K, et al The use of transcranial ultrasonography to predict stroke in sickle cell disease. The New England journal of medicine. 1992;326(9):605–10. 10.1056/NEJM199202273260905 . 1734251

[11] Adams RJ, McKie VC, Carl EM, Nichols FT, Perry R, Brock K, et al Long-term stroke risk in children with sickle cell disease screened with transcranial Doppler. Annals of neurology. 1997;42(5):699–704. 10.1002/ana.410420505 . 9392568

[12] Vollmer WM, Tsai R, Wu Y, Li YH, Johnson LR, Williams OD, et al Patterns of lung function in asymptomatic nonsmoking men and women in the People's Republic of China. Annals of epidemiology. 2002;12(5):295–302. . 12062915 10.1016/s1047-2797(01)00288-5

[13] Nishida M, Moriyama T, Ishii K, Takashima S, Yoshizaki K, Sugita Y, et al Effects of IL-6, adiponectin, CRP and metabolic syndrome on subclinical atherosclerosis. Clinica chimica acta; international journal of clinical chemistry. 2007;384(1–2):99–104. 10.1016/j.cca.2007.06.009 . 17618612

[14] Xie G, Laskowitz DT, Turner EL, Egger JR, Shi P, Ren F, et al Baseline health-related quality of life and 10-year all-cause mortality among 1739 Chinese adults. PloS one. 2014;9(7):e101527 10.1371/journal.pone.0101527 25007092 PMC4090174

[15] International Labour Organisation. International Standard Classification of Occupations (ISCO-88). Geneva 1988.

[16] Zhang P, Huang Y, Li Y, Shi P, Lu M, Detrano R, et al Gender and risk factor dependence of cerebral blood flow velocity in Chinese adults. Brain research bulletin. 2006;69(3):282–7. 10.1016/j.brainresbull.2005.12.008 . 16564423

[17] Zhang P, Huang Y, Li Y, Lu M, Wu Y. A large-scale study on relationship between cerebral blood flow velocity and blood pressure in a natural population. Journal of human hypertension. 2006;20(10):742–8. 10.1038/sj.jhh.1002068 . 16810278

[18] Gao S, Lam WW, Chan YL, Liu JY, Wong KS. Optimal values of flow velocity on transcranial Doppler in grading middle cerebral artery stenosis in comparison with magnetic resonance angiography. Journal of neuroimaging: official journal of the American Society of Neuroimaging. 2002;12(3):213–8. . 12116738

[19] Hwang CS, Shau WY, Tegeler CH. Doppler velocity criteria based on receiver operating characteristic analysis for the detection of threshold carotid stenoses. Journal of neuroimaging: official journal of the American Society of Neuroimaging. 2002;12(2):124–30. . 11977906 10.1111/j.1552-6569.2002.tb00108.x

[20] Huang YN, Gao S, Li SW, Huang Y, Li JF, Wong KS, et al Vascular lesions in Chinese patients with transient ischemic attacks. Neurology. 1997;48(2):524–5. . 9040750 10.1212/wnl.48.2.524

[21] Sacco RL, Kargman DE, Gu Q, Zamanillo MC. Race-ethnicity and determinants of intracranial atherosclerotic cerebral infarction. The Northern Manhattan Stroke Study. Stroke; a journal of cerebral circulation. 1995;26(1):14–20. . 7839388 10.1161/01.str.26.1.14

[22] Ni J, Yao M, Gao S, Cui LY. Stroke risk and prognostic factors of asymptomatic middle cerebral artery atherosclerotic stenosis. Journal of the neurological sciences. 2011;301(1–2):63–5. 10.1016/j.jns.2010.10.029 . 21094496

[23] Zhang S, Zhou Y, Zhang Y, Gao X, Zhang Q, Wang A, et al Prevalence and risk factors of asymptomatic intracranial arterial stenosis in a community-based population of Chinese adults. European journal of neurology: the official journal of the European Federation of Neurological Societies. 2013;20(11):1479–85. 10.1111/ene.12210 . 23746073

[24] Liu CY, Chen CQ. Intra- and extracranial atherosclerotic stenosis in China: epidemiology, diagnosis, treatment and risk factors. European review for medical and pharmacological sciences. 2014;18(22):3368–79. . 25491610

[25] Babikian VL, Feldmann E, Wechsler LR, Newell DW, Gomez CR, Bogdahn U, et al Transcranial Doppler ultrasonography: year 2000 update. Journal of neuroimaging: official journal of the American Society of Neuroimaging. 2000;10(2):101–15. . 10800264 10.1111/jon2000102101

[26] Sada S, Reddy Y, Rao S, Alladi S, Kaul S. Prevalence of middle cerebral artery stenosis in asymptomatic subjects of more than 40 years age group: a transcranial Doppler study. Neurology India. 2014;62(5):510–5. 10.4103/0028-3886.144443 . 25387620

[27] Wong KS, Ng PW, Tang A, Liu R, Yeung V, Tomlinson B. Prevalence of asymptomatic intracranial atherosclerosis in high-risk patients. Neurology. 2007;68(23):2035–8. 10.1212/01.wnl.0000264427.09191.89 . 17548555

[28] Huang HW, Guo MH, Lin RJ, Chen YL, Luo Q, Zhang Y, et al Prevalence and risk factors of middle cerebral artery stenosis in asymptomatic residents in Rongqi County, Guangdong. Cerebrovascular diseases. 2007;24(1):111–5. 10.1159/000103125 . 17519553

[29] Wong KS, Huang YN, Yang HB, Gao S, Li H, Liu JY, et al A door-to-door survey of intracranial atherosclerosis in Liangbei County, China. Neurology. 2007;68(23):2031–4. 10.1212/01.wnl.0000264426.63544.ee . 17548554

[30] Gaciong Z, Sinski M, Lewandowski J. Blood pressure control and primary prevention of stroke: summary of the recent clinical trial data and meta-analyses. Current hypertension reports. 2013;15(6):559–74. 10.1007/s11906-013-0401-0 24158454 PMC3838588

[31] Huang Y, Cai X, Li Y, Su L, Mai W, Wang S, et al Prehypertension and the risk of stroke: a meta-analysis. Neurology. 2014;82(13):1153–61. 10.1212/WNL.0000000000000268 . 24623843

[32] Lackland DT, Roccella EJ, Deutsch AF, Fornage M, George MG, Howard G, et al Factors influencing the decline in stroke mortality: a statement from the American Heart Association/American Stroke Association. Stroke; a journal of cerebral circulation. 2014;45(1):315–53. 10.1161/01.str.0000437068.30550.cf . 24309587 PMC5995123

[33] Tse LA, Fang XH, Wang WZ, Qiu H, Yu IT. Incidence of ischaemic and haemorrhagic stroke and the association with smoking and smoking cessation: a 10-year multicentre prospective study in China. Public health. 2012;126(11):960–6. 10.1016/j.puhe.2012.07.010 . 23062630

[34] Gu D, Gupta A, Muntner P, Hu S, Duan X, Chen J, et al Prevalence of cardiovascular disease risk factor clustering among the adult population of China: results from the International Collaborative Study of Cardiovascular Disease in Asia (InterAsia). Circulation. 2005;112(5):658–65. 10.1161/CIRCULATIONAHA.104.515072 . 16043645

