# Supplementary material for: Peak Systolic Velocity Measurements with Transcranial Doppler Ultrasound Is a Predictor of Incident Stroke among the General Population in China
Source: PLoS One. 2016 Aug 11;11(8):e0160967. doi: 10.1371/journal.pone.0160967 (PMC4981305; doi:10.1371/journal.pone.0160967)
Supplement: S1 Table — Hypertension was defined as a mean SBP of ≥140 mm Hg or a mean DBP of ≥90 mm Hg, or the use of antihypertensive drugs in the past 2 weeks. (DOCX) [file pone.0160967.s002.docx]

S1Table. The relationship between frequency of physical exercise and age, hypertension

| Frequency of physical exercise | | Almost every day | 1-4 times per week | Hardly ever | P value |
| --- | --- | --- | --- | --- | --- |
| Age (Yrs) | <50 | 123 (33.5) | 42 (11.4) | 202 (55.0) | <.0001 |
|  | 50-59 | 275 (58.4) | 29 (6.2) | 167 (35.5) |  |
|  | ≥60 | 272 (64.0) | 19 (4.5) | 134 (31.5) |  |
| Hypertension ^£^ | No | 325 (48.9) | 47 (7.1) | 292 (44.0) | 0.0052 |
|  | Yes | 345 (57.6) | 43 (7.2) | 211 (35.2) |  |

^£^ Hypertension was defined as a mean SBP of ≥140 mm Hg or a mean DBP of ≥90 mm Hg, or the use of antihypertensive drugs in the past 2 weeks.
